# Performance Enhancement for Full-Duplex Relaying with Time-Switching-Based SWIPT in Wireless Sensors Networks

**DOI:** 10.3390/s21113847

**Published:** 2021-06-02

**Authors:** Phu Tran Tin, Tan N. Nguyen, Dinh-Hieu Tran, Miroslav Voznak, Van-Duc Phan, Symeon Chatzinotas

**Affiliations:** 1 Faculty of Electronics Technology, Industrial University of Ho Chi Minh City, Ho Chi Minh City 700000, Vietnam; phutrantin@iuh.edu.vn; 2 Wireless Communications Research Group, Faculty of Electrical and Electronics Engineering, Ton Duc Thang University, Ho Chi Minh City 700000, Vietnam; 3 Interdisciplinary Centre for Security, Reliability and Trust (SnT), The University of Luxembourg, Luxembourg L-1111, Luxembourg; hieu.tran-dinh@uni.lu (D.-H.T.); symeon.chatzinotas@uni.lu (S.C.); 4 Faculty of Electrical Engineering and Computer Science, VSB-Technical University of Ostrava, 17. Listopadu 2172/15, 708 00 Ostrava, Czech Republic; miroslav.voznak@vsb.cz; 5 Faculty of Automobile Technology, Van Lang University, Ho Chi Minh City 700000, Vietnam; duc.pv@vlu.edu.vn

**Keywords:** decode-and-forward (DF), energy harvesting, full-duplex, outage probability, SWIPT, time-switching relaying (TSR)

## Abstract

Full-duplex (FD) with simultaneous wireless information and power transfer (SWIPT) in wireless ad hoc networks has received increased attention as a technology for improving spectrum and energy efficiency. This paper studies the outage performance for a SWIPT-based decode-and-forward (DF) FD relaying network consisting of a single-antenna source S, a two-antenna relay R, and a multi-antenna destination D. Specifically, we propose four protocols, namely static time-switching factor with selection combining (STSF-SC), static time-switching factor with maximal ratio combining (STSF-MRC), optimal dynamic time-switching factor with selection combining (ODTSF-SC), and optimal dynamic time-switching factor with maximal ratio combining (ODTSF-MRC) to fully investigate the outage performance of the proposed system. In particular, the optimal time-switching factor from the ODTSF-SC and ODTSF-MRC methods is designed to maximize the total received data at the destination. In this context, we derive exact closed-formed expressions for all schemes in terms of the outage probability (OP). Finally, the Monte Carlo simulations are conducted to corroborate the theoretical analysis’s correctness and the proposed schemes’ effectiveness.

## 1. Introduction

The Internet of Things (IoT) has played a key role in fifth generation (5G) and beyond due to its ability to improve human quality of life such as health care, wearable devices, smart cities, agriculture, industrial automation, intelligent street lighting, maintenance management, etc. [1,2,3,4]. The number of IoT devices (IoTDs) is forecast to reach 10.07 and 25.44 in 2021 and 2030, respectively [5]. Nevertheless, the unprecedented increase of IoTDs will lead to two fundamental challenges in IoT networks, i.e., large energy supply sources and spectrum scarcity. The IoTDs are usually equipped with batteries with limited energy capacity. In particular, the problem of improving energy budget for IoTDs is more urgent in wireless ad hoc networks since the IoTDs are self-configuring, self-organizing, and without human intervention [6]. Specifically, in the case that IoTDs are randomly deployed in inaccessible areas, they should cooperate with other ones within their range to implement the task of monitoring or to observe the target scene and to communicate with the base station that collects information from all IoTDs [7]. Consequently, energy harvesting (EH) has emerged as a promising solution to prolong the lifetime of IoTDs in wireless ad hoc networks. Potential ambient energy sources encompass solar [8], wind, and water [9,10]. In particular, radio frequency (RF) EH has received significant attention from researchers since it does not depend on the randomness and intermittency of the surrounding environments, i.e., wind and solar [2,11,12,13,14,15,16,17]. Lee et al. [12] investigated the impact of RF EH in cognitive radio networks (CRNs). Specifically, the secondary transmitters (STs) could harvest energy from primary transmitters (PTs) and stored it in their rechargeable batteries. Then, the STs used this energy for data transmission when the PTs were far away. Maso et al. [13] introduced a new energy-recycling FD architecture, in which a three-port element consisting of an energy harvester a power divider is added between the receiver (RX) chain and the circulator. This new element improved the state-of-the-art self-interference cancellation and provided both spectral and energy efficiency. Tan et al. [14] investigated the EH-based two-way relay selection in wireless ad hoc networks. In the system model, two sources could communicate with each other through the help of multiple relays using three-phase digital network coding. Specifically, the relay harvested energy from two sources using the power-splitting (PS) method in the first and second phases. Then, it transmitted data to sources in the third phase by leveraging the harvested energy. Hieu et al. [15] studied the physical layer security (PLS) of a multi-hop multi-path wireless sensor network (WSN), whereas source and relay nodes can harvest energy from a power beacon to transfer data to the destination. In [2,16], the authors investigated the wireless power transfer (WPT) for backscatter communication (BackCom). Kellogg et al. [16] proposed a novel communication system that bridged RF-powered devices, i.e., Wifi-Backscatter tags, to the Internet system through Wifi-infrastructure. In particular, the Wifi-Backscatter tags did not need to be equipped with batteries since they could harvest energy from ambient RF sources such as TV, Wifi, and cellular networks. In [2], the authors investigated a backscatter-assisted data offloading in OFDMA-based wireless-powered (WP) MEC for IoT systems. Specifically, backscatter-assisted IoT users (BUs) harvested energy from the ground gateway (GW)’s RF signals using the time-switching method. Then, they used the harvested energy to offload computation tasks to the GW.

As RF signals can carry both energy and information simultaneously, a concept of simultaneous wireless information and power transfer (SWIPT) was introduced by Varshney in 2008 [18]. Instead of considering flat fading as in [18], Grover [19] has developed the work [18] to a frequency-selective fading channel. Nevertheless, the works [18,19] assumed that an ideal receiver could decode energy and signal simultaneously, which is impractical. Therefore, Zhang et al. [20] introduced two different types of receivers, i.e., separated between information and energy receivers and co-located receivers. Furthermore, they proposed two practical designs for a co-located scenario, namely time-switching (TS) and power-splitting (PS).

Based on the TS and PS methods in [20], Nasir et al. proposed two relaying protocols, namely time-switching-based relaying (TSR) and power-splitting-based relaying (PSR), to apply SWIPT at the relay node. The SWIPT-based cooperative relaying networks have been received significant attention from researchers [21,22,23,24,25,26,27]. In [21], the authors investigated physical layer security (PLS) for a two-way relay network using hybrid time-switching and power-splitting (HTPSR) protocol. Furthermore, they derived the exact closed-form expressions of the intercept probability (IP) at the eavesdropper, the outage probability (OP) at the source nodes, the average secrecy capacity (ASC), and the secrecy outage probability (SOP). Nguyen et al. [22] proposed a novel two-way DF relaying network over Rician channels. Moreover, analytical expressions in terms of OP, throughput, and ergodic capacity were derived and demonstrated. Yuan et al. [23] studied the throughput and outage performance of a SWIPT decode-and-forward (DF) relaying system with non-linear EH model. Nguyen et al. [24] proposed a novel system model in which a relay node can receive both information and energy from two sources applying HTPSR. For performance investigation, they adopted three relaying methods, namely decode-and-forward (DF), amplify-and-forward (AF), and hybrid decode-and-forward (HDAF). In [25,26,27], the authors studied the SWIPT technique in wireless ad hoc networks. Zhou et al. [25] investigated the outage performance of maximum ratio transmission (MRT) in ad hoc networks with SWIPT. In their system model, they assumed that transmitters are equipped with multiple antennas using MRT and receivers had single antenna with energy circuit. Psomas et al. [26] studied the successive interference cancellation (SIC) technique from a SWIPT standpoint in bipolar ad hoc networks. Specifically, we showed how each receiver can use SIC to boost the WPT without affecting the information decoding. Park et al. [27] considered a SWIPT-enabled ad hoc network in which separated or co-located energy transmitting access points (EAPs)/information transmitting APs (IAPs) can transfer energy as well as information using a common spectrum, and receivers can decode the incoming data and/or harvest the RF energy.

All the works mentioned above for SWIPT-enabled cooperative relaying networks are limited for half-duplex transmission, and thus two time slots are required to transmit the information from source to destination. Thanks to the development of self-interference cancellation (SIC) techniques which can achieve high SI reduction, full-duplex (FD) cooperative communications (CC) technology becomes one of a critical core technique for fifth generation and beyond [28,29]. Full-duplex radio has great applications for wireless communications, not least because it can save the spectrum needs by half while still guaranteeing the same system performance [30,31,32,33,34]. In [30], the authors analyzed the outage performance of a novel system model that considered EH, FD, and cooperative non-orthogonal multiple access (NOMA). Using the amplify-and-forward protocol, they derived the expressions of the OP, throughput, and the optimal value of the time-switching factor to obtain the best performance. References [31,32] considered FD relaying for unmanned aerial vehicle (UAV) communications. Ye et al. [31] explored the rotary-wing UAV-enabled FD wireless-powered IoT networks, in which a UAV serves multiple energy-constrained IoTDs. In this context, they proposed three optimization problems, namely sum-throughput maximization (STM), total-time minimization (TTM), and total-energy minimization (TEM) optimization problems. In [32], a FD-UAV relay was deployed to improve the data transmission rate of millimeter-wave (mmWave) networks. Specifically, they aimed to maximize the achievable rate from source to destination by jointly optimizing the UAV trajectory, analog beamforming, and power allocation. In contrast to [30,31,32] that only considered Raleigh fading channel, [33,34] took into account the Rician fading model. In [14], a new system model of DF FD relaying network over the Rician fading environment was proposed and analyzed. Ding et al. [34] analyzed the achievable rate performance of a multi-user FD massive multiple-input multiple-output (MIMO) system with low-resolution analog-to-digital converters (ADCs) and digital-to-analog converters (DACs) using Rician fading channels.

Although the SWIPT and FD techniques are promising solutions for improving energy and spectrum efficiency, the study of FD- and SWIPT-enabled cooperative relaying networks is still limited. In the literature, references [35,36] considered a similar system model as our paper. Nevertheless, they did not consider the SWIPT technique, and the destination was only equipped with one antenna. To overcome the limitations in [35,36], reference [37] studied SWIPT with FD for a dual-hop cooperative network with one source, one FD relay, and one destination. However, our designed model investigates a single-input-multiple-output (SIMO) system instead of SISO model in [37]. Furthermore, we adopt optimal TS factor-based method that is not studied in [37]. Furthermore, another study in [38] also has a similar system design to our work. Specifically, they investigated the outage performance of a FD wireless-powered DF relaying protocol with self-energy-recycling (SER). Nevertheless, their considered system model is totally different from our work since they only have a single-antenna destination. Moreover, the relay node in our paper also performs FD mode but without SER since the harvested energy from the loop-back channel is assumed to be very small and can be neglectable. Motivated by the above discussions, this paper proposes and investigates the system performance analysis of the DF FD scheme for the TS-based SWIPT network. The main contributions of this research are summarized as follows:

- We propose a SIMO system model in which a two-antenna FD relay node can harvest energy from RF signals of a single-antenna source and then use the harvested energy to transfer information to a multi-antenna destination. Specifically, the destination can adopt SC or MRC to decode the received information.
- Both static TS factor (STSF)- and optimal dynamic TS factor (ODTSF)-based methods are investigated in our work. Especially in ODTSF methods, we derive the exact closed form of optimal dynamic TS factor for maximizing the total throughput obtained at the destination.
- To fully investigate the system performance, we propose four protocols, namely static time-switching factor with selection combining (STSF-SC), static time-switching factor with maximal ratio combing (STSF-MRC), optimal dynamic time-switching factor with selection combining (ODTSF-SC), and optimal dynamic time-switching factor with maximal ratio combing (ODTSF-MRC).
- Monte Carlo simulations are performed to corroborate the effectiveness of our proposed methods. Specifically, the results show that ODTSF-MRC always outperforms other schemes in terms of outage probability.

The rest of this paper is organized as follows. The system model of the proposed protocol is described in Section 2. In Section 3, performance evaluation is performed. The simulation results are shown in Section 4. Finally, this paper is concluded in Section 5.

## 2. System Model

**Figure 1:**
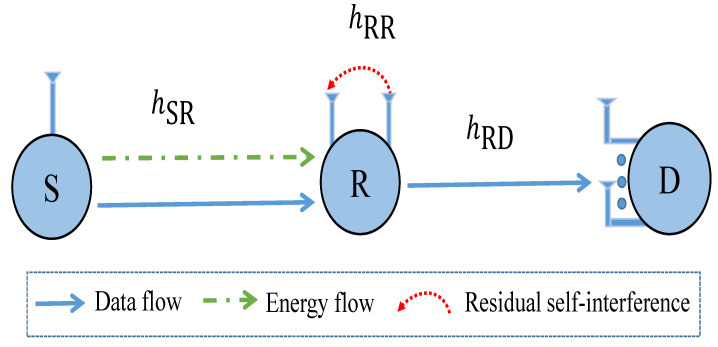
System model.

**Figure 2:**
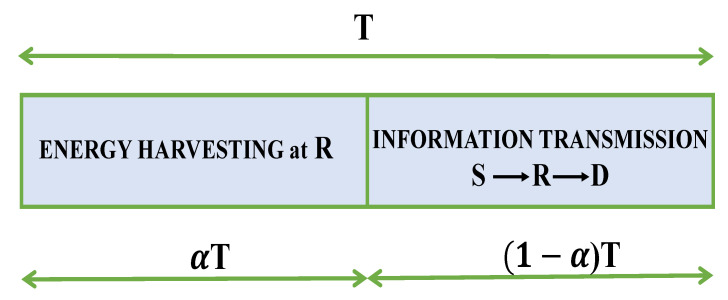
Schematic illustration of EH and IT processes with a time-switching relaying protocol at the relay.

As shown in Figure 1, we consider a full-duplex and SWIPT-assisted relaying system in ad hoc wireless networks, where a source S attempts to transmit data to the destination D with the help of one relay R. Assume that S, R, and D are equipped with single, double, and *M* antennas. Furthermore, S and D operate on half-duplex mode while relay R can work on full-duplex one. Due to physical isolation between the source and destination or severe fading, the direct link between a source and a destination is missing. Thus, a relay is used to convey information from a source to a destination. For simplicity of implementation, the DF relaying method and time-switching architecture are adopted at the relay R. Specifically, the relay R decodes the packet received from the source S by fully decoding, and then encodes and forwards the packet to the destination D. The relay R is assumed to be an energy-constrained or energy-selfish device, and it can harvest energy from source’s RF signals using time-switching-based method since it is equipped with an energy harvester circuit. In Figure 2, the data transmission is realized over two time slots. Specifically, a time divider is used to divide the input signals into two portions: a part of time slot αT is designed for energy harvesting, and the second part (1−α)T is designed for information transmission. Herein, α∈[0,1] is the time-switching factor, with α equals zero/one means that all received signals is dedicated for data transmission/energy harvesting.

For ease of presentation, let us denote $P_{S}$, $P_{R}$, and $N_{0}$ as the transmit power of source, relay, and the variance of additive noises at receivers, respectively. Furthermore, let us denote $h_{\mathrm{SR}}$, $h_{{\mathrm{RD}}_{m}}$, and $h_{\mathrm{RR}}$ as the channel coefficients of the links between S→R, $R\to D_{m}$, and loop-back interference of the relay R, respectively. We assume that channels follow independent and identically Rayleigh distribution (i.i.d.). Consequently, the channel gains $|h_{\mathrm{SR}}{|}^{2}$, $|h_{{\mathrm{RD}}_{m}}{|}^{2}$, and $|h_{\mathrm{RR}}{|}^{2}$ follow exponential distribution with rate parameters $\lambda_{\mathrm{SR}}$, $\lambda_{\mathrm{RD}}$, and $\lambda_{\mathrm{RR}}$, respectively.

### 2.1. Energy Harvesting and Information Transmission

The received signal at the relay can be expressed as

(1) $$\begin{array}{c} y_{R}=h_{\mathrm{SR}}x_{S}+h_{\mathrm{RR}}x_{R}+n_{R}, \end{array}$$

where $x_{S}$ is the transmitted signal at the source S and $E\{|x_{S}{|}^{2}\}=P_{S}$, with $E\left\{•\right\}$ is the expectation operator, $P_{S}$ is the average transmit power at the bth source; $x_{R}$ is the self-interference since the relay R operates at the full-duplex mode and $E\{|x_{R}{|}^{2}\}=P_{R}$; $n_{R}$ is the zero mean additive white Gaussian noise (AWGN) with variance $N_{0}$; $h_{\mathrm{SR}}$ is the channel coefficient from S→R; $h_{\mathrm{RR}}$ is the channel coefficient due to loop-back interference at relay R.

At the first time slot, the harvested energy at the relay can be calculated as

(2) $$\begin{array}{c} E_{R}=\eta \alpha TP_{S}{\left|h_{\mathrm{SR}}\right|}^{2}, \end{array}$$

where 0≤η≤1 is the energy conversion efficient and α is the time-switching factor.

From (2), the transmit power of the relay R can be given by

(3) $$\begin{array}{c} P_{R}=\frac{E_{R}}{(1-\alpha )T}=\frac{\eta \alpha P_{S}{\left|h_{\mathrm{SR}}\right|}^{2}}{\left(1-\alpha \right)}=\mu P_{S}{\left|h_{\mathrm{SR}}\right|}^{2}, \end{array}$$

where $\mu ≜\frac{\eta \alpha }{\left(1-\alpha \right)}$.

In this work, we adopt decode-and-forward (DF) relaying technique. Thus, the signal-to-interference-plus-noise-ratio (SINR) at the relay R can be given by

(4) $$\begin{array}{c} \gamma_{R}=\frac{P_{S}{\left|h_{\mathrm{SR}}\right|}^{2}}{P_{R}{\left|h_{\mathrm{RR}}\right|}^{2}+N_{0}}. \end{array}$$

By substituting (4) into (3) and using the fact that $N_{0}\ll P_{S}$, it yields

(5) $$\begin{array}{c} \gamma_{R}=\frac{P_{S}{\left|h_{\mathrm{SR}}\right|}^{2}}{\mu P_{S}|h_{\mathrm{SR}}{|}^{2}{\left|h_{\mathrm{RR}}\right|}^{2}+N_{0}}\approx \frac{1}{\mu |h_{\mathrm{RR}}{|}^{2}}. \end{array}$$

**Remark** **1.** *In this paper, both selection combining (SC) and maximal ratio combining (MRC) techniques are considered at the destination to investigate each one’s benefits.*

#### 2.1.1. Case 1: Selection Combining (SC) Is Adopted at the Destination

The received signal at *m*-th antenna of the destination is given by

(6) $$\begin{array}{c} y_{D_{m}}^{\mathrm{SC}}={\left|h_{\mathrm{SR}}\right|}^{2}x_{R}+n_{D_{m}}, \end{array}$$

where $n_{D_{m}}$ is the AWGN with variance $N_{0}$.

From (6), the signal-to-noise-ratio (SNR) at the destination can be obtained by

(7) $$\begin{array}{c} \gamma_{D_{m}}^{\mathrm{SC}}=\frac{|h_{{\mathrm{RD}}_{m}}{|}^{2}P_{R}}{N_{0}}=\frac{\mu P_{S}|h_{\mathrm{SR}}{|}^{2}{\left|h_{{\mathrm{RD}}_{m}}\right|}^{2}}{N_{0}}=\mu \Phi |h_{\mathrm{SR}}{|}^{2}{\left|h_{{\mathrm{RD}}_{m}}\right|}^{2}, \end{array}$$

where $\Phi ≜\frac{P_{S}}{N_{0}}$.

**Remark** **2.** *In SC technique, we propose the optimal antenna selection protocol at the destination in which the best one is selected as follows*

(8) $$\begin{array}{c} m^{⋆}=\arg\max_{1\leq m\leq M}{\left|h_{{\mathrm{RD}}_{m}}\right|}^{2}. \end{array}$$

By denoting $X=\max_{m=1,2,\ldots ,M}\left({\left|h_{{\mathrm{RD}}_{m}}\right|}^{2}\right)$, the cumulative distribution function (CDF) of *X* can be given by

(9) $$\begin{array}{c} F_{X}\left(x\right)=\sum_{m=0}^{M}{\left(-1\right)}^{m}C_{M}^{m}e^{-\lambda_{\mathrm{RD}}mx}=1+\sum_{m=1}^{M}{\left(-1\right)}^{m}C_{M}^{m}e^{-\lambda_{\mathrm{RD}}mx}, \end{array}$$

where $C_{M}^{m}=\frac{M!}{m!(M-m)!}$ and $|h_{{\mathrm{RD}}_{m}}{|}^{2}$ is exponentially distributed with parameter $\lambda_{\mathrm{RD}}$.

Then, the probability density function (PDF) of random variable (RV) *X* can be expressed as

(10) $$\begin{array}{c} f_{X}\left(x\right)=\lambda_{{\mathrm{RD}}_{m}}\sum_{m=0}^{M-1}{\left(-1\right)}^{m}C_{M-1}^{m}e^{-\lambda_{\mathrm{RD}}\left(m+1\right)x}. \end{array}$$

#### 2.1.2. Case 2: Maximal Ratio Combining (MRC) Is Adopted at the Destination

Since the destination adopt MRC technique, it can incorporate information from all antennas. Thus, received signals at the destination is given by

(11) $$\begin{array}{c} y_{D}^{\mathrm{MRC}}=\sum_{m=1}^{M}{\left|h_{{\mathrm{RD}}_{m}}\right|}^{2}x_{R}+n_{D}. \end{array}$$

By applying (3) and (7), the SNR at the destination can be represented as

(12) $$\begin{array}{c} \gamma_{D}^{\mathrm{MRC}}=\frac{\sum_{m=1}^{M}{\left|h_{{\mathrm{RD}}_{m}}\right|}^{2}P_{R}}{N_{0}}=\frac{\mu P_{S}|h_{\mathrm{SR}}{|}^{2}\sum_{m=1}^{M}{\left|h_{{\mathrm{RD}}_{m}}\right|}^{2}}{N_{0}}=\mu \Phi |h_{\mathrm{SR}}{|}^{2}\sum_{m=1}^{M}{\left|h_{{\mathrm{RD}}_{m}}\right|}^{2}, \end{array}$$

By denoting $Y=\sum_{m=1}^{M}{\left|h_{{\mathrm{RD}}_{m}}\right|}^{2}$, the PDF of *Y* can be given by [39]

(13) $$\begin{array}{c} f_{Y}\left(y\right)=\frac{{\left(\lambda_{\mathrm{RD}}\right)}^{M}}{(M-1)!}y^{M-1}e^{-\lambda_{\mathrm{RD}}y}. \end{array}$$

## 3. Outage Probability (OP) Analysis

### 3.1. Case 1: Static Time-Switching Factor (STSF)

In the first scenario, we investigate the OP with a fixed value of time-switching factor. This method’s advantage is simplicity since we can directly obtain the OP corresponding to each time-switching value.

#### 3.1.1. With SC

The outage probability can be formulated as follows [40]

(14) $$\begin{array}{c} {\mathrm{OP}}_{\mathrm{SC}}=Pr\left(\gamma_{e2e}^{\mathrm{SC}}<\gamma_{\mathrm{th}}\right), \end{array}$$

where $\gamma_{e2e}^{SC}≜\min\left(\gamma_{R},\gamma_{D_{m}}^{\mathrm{SC}}\right)$ and $\gamma_{\mathrm{th}}$ is the SNR threshold of the system.

**Theorem** **1.** *In static time-switching factor scheme with SC, the closed-form expression of OP at the destination D can be expressed as*

(15) $$\begin{array}{cc} {\mathrm{OP}}_{\mathrm{SC}} & =1-\left(1-\exp\left(-\frac{\lambda_{\mathrm{RR}}}{\mu \gamma_{\mathrm{th}}}\right)\right)\left(2\sum_{m=0}^{M-1}{\left(-1\right)}^{m}C_{M-1}^{m}M\sqrt{\frac{\chi_{2}\gamma_{\mathrm{th}}}{\mu }}K_{1}\left(2\sqrt{\frac{\chi_{1}\gamma_{\mathrm{th}}}{\mu }}\right)\right), \end{array}$$

*where $\chi_{1}≜\frac{\lambda_{\mathrm{SR}}\lambda_{\mathrm{RD}}\left(m+1\right)}{\Phi }$, $\chi_{2}≜\frac{\lambda_{\mathrm{SR}}\lambda_{\mathrm{RD}}}{\Phi (m+1)}$.*

**Proof.**  By substituting (5) and (7) into (14), the OP can be calculated as

(16) $$\begin{array}{cc} {\mathrm{OP}}_{\mathrm{SC}} & =Pr\left[\left(\min\left(\frac{1}{\mu |h_{\mathrm{RR}}{|}^{2}},\mu \Phi {\left|h_{\mathrm{SR}}\right|}^{2}\max\left(|h_{RD_{m}}{|}^{2}\right)\right)\right)<\gamma_{\mathrm{th}}\right]\\ \; & =Pr\left[\left(\min\left(\frac{1}{\mu Z},\mu \Phi XT\right)\right)<\gamma_{\mathrm{th}}\right]=1-\underset{P_{1}}{\underbrace{Pr\left(\frac{1}{\mu Z}\geq \gamma_{\mathrm{th}}\right)}}\underset{P_{2}}{\underbrace{Pr\left(\mu \Phi XT\geq \gamma_{\mathrm{th}}\right)}}, \end{array}$$

where $Z≜|h_{\mathrm{RR}}{|}^{2},$ $X≜\max\left(|h_{RD_{m}}{|}^{2}\right)$, and $T≜|h_{\mathrm{SR}}{|}^{2}$.

Herein, $P_{1}$ in (16) can be calculated as

(17) $$\begin{array}{c} P_{1}=Pr\left(\frac{1}{\mu Z}\geq \gamma_{\mathrm{th}}\right)\;=\;Pr\left(Z\geq \frac{1}{\mu \gamma_{\mathrm{th}}}\right)\;=\;1-e^{-\frac{\lambda_{\mathrm{RR}}}{\mu \gamma_{\mathrm{th}}}}. \end{array}$$

Next, $P_{2}$ in (16) can be calculated as

(18) $$\begin{array}{cc} P_{2} & =Pr\left(\mu \Phi XT\geq \gamma_{\mathrm{th}}\right)\;=\;1-Pr\left(\mu \Phi XT<\gamma_{\mathrm{th}}\right)\;=\;1-Pr\left(T<\frac{\gamma_{\mathrm{th}}}{\mu \Phi X}\right)\\ \; & =1-\int_{0}^{+\infty }F_{T}\left(\frac{\gamma_{\mathrm{th}}}{\mu \Phi x}\right)f_{X}\left(x\right)dx, \end{array}$$

By substituting (10) into (16), we have

(19) $$\begin{array}{cc} P_{2} & =\sum_{m=0}^{M-1}{\left(-1\right)}^{m}C_{M-1}^{m}\int_{0}^{+\infty }\lambda_{\mathrm{RD}}e^{-\lambda_{\mathrm{RD}}\left(m+1\right)x-\frac{\lambda_{\mathrm{SR}}\gamma_{\mathrm{th}}}{\mu \Phi x}}dx, \end{array}$$

By applying [41], $P_{2}$ can be reformulated as

(20) $$\begin{array}{c} P_{2}=2\sum_{m=0}^{M-1}{\left(-1\right)}^{m}C_{M-1}^{m}\sqrt{\frac{\chi_{2}\gamma_{\mathrm{th}}}{\mu }}K_{1}\left(2\sqrt{\frac{\chi_{1}\gamma_{\mathrm{th}}}{\mu }}\right), \end{array}$$

where $K_{v}\left\{•\right\}$ is the modified Bessel function of second kind with vth order.

Then, by substituting (17) and (20) into (16), we can obtain (15), which finishes the proof.  □

#### 3.1.2. With MRC

**Theorem** **2.** *In static time-switching factor scheme with MRC, the closed-form expression of OP at the destination D can be expressed as*

(21) $$\begin{array}{c} {\mathrm{OP}}_{\mathrm{MRC}}=1-\left(1-\exp\left(-\frac{\lambda_{\mathrm{RR}}}{\mu \gamma_{\mathrm{th}}}\right)\right)\left\{\frac{2}{(M-1)!}{\left(\frac{\chi_{3}\gamma_{\mathrm{th}}}{\mu }\right)}^{M/2}K_{M}\left(2\sqrt{\frac{\chi_{3}\gamma_{\mathrm{th}}}{\mu }}\right)\right\}, \end{array}$$

*where $\chi_{3}≜\frac{\lambda_{\mathrm{SR}}\lambda_{\mathrm{RD}}}{\Phi }$.*

**Proof.**  Similar to (14), the outage probability of MRC ${\mathrm{OP}}_{\mathrm{MRC}}$ is given by

(22) $$\begin{array}{cc} {\mathrm{OP}}_{\mathrm{MRC}} & =Pr\left(\gamma_{e2e}^{\mathrm{MRC}}<\gamma_{\mathrm{th}}\right)\;=\;Pr\left\{\left(\min\left(\frac{1}{\mu {\left|h_{\mathrm{RR}}\right|}^{2}},\mu \Phi {\left|h_{\mathrm{SR}}\right|}^{2}\sum_{m=1}^{M}{\left|h_{RD_{m}}\right|}^{2}\right)\right)<\gamma_{\mathrm{th}}\right\}\\ & =Pr\left\{\left(\min\left(\frac{1}{\mu Z},\mu \Phi TY\right)\right)<\gamma_{\mathrm{th}}\right\} \end{array}$$

where $\gamma_{e2e}^{\mathrm{MRC}}=\min\left(\gamma_{R},\gamma_{D}^{\mathrm{MRC}}\right)$.

For ease of analysis, ${\mathrm{OP}}_{\mathrm{MRC}}$ is rewritten as

(23) $$\begin{array}{c} {\mathrm{OP}}_{\mathrm{MRC}}=1-\underset{P_{1}}{\underbrace{Pr\left(\frac{1}{\mu Z}\geq \gamma_{\mathrm{th}}\right)}}\underset{P_{3}}{\underbrace{Pr\left(\mu \Phi TY\geq \gamma_{\mathrm{th}}\right)}}, \end{array}$$

It is easy to see that $P_{1}$ was determined as in (17). Then, $P_{3}$ can be computed by

(24) $$\begin{array}{c} P_{3}=Pr\left(\mu \Phi TY\geq \gamma_{\mathrm{th}}\right)\;=\;1-\int_{0}^{+\infty }F_{T}\left(\frac{\gamma_{\mathrm{th}}}{\mu \Phi y}\right)f_{Y}\left(y\right)dy, \end{array}$$

By substituting (13) into (24), we have

(25) $$\begin{array}{c} P_{3}=\int_{0}^{+\infty }\frac{{\left(\lambda_{\mathrm{RD}}\right)}^{M}}{(M-1)!}y^{M-1}e^{-\lambda_{\mathrm{RD}}y-\frac{\lambda_{\mathrm{SR}}\gamma_{\mathrm{th}}}{\mu \Phi y}}dy, \end{array}$$

By adopting [41], $P_{3}$ can be reformulated as

(26) $$\begin{array}{c} P_{3}=\frac{2}{(M-1)!}{\left(\frac{\chi_{3}\gamma_{\mathrm{th}}}{\mu }\right)}^{M/2}K_{M}\left(2\sqrt{\frac{\chi_{3}\gamma_{\mathrm{th}}}{\mu }}\right). \end{array}$$

By substituting (17) and (26) into (23), ${\mathrm{OP}}_{\mathrm{MRC}}$ can be obtained as in (21). □

### 3.2. Case 2: Optimal Dynamic Time-Switching Factor (ODTSF)

In the second scenario, we find the optimal $\alpha^{⋆}$ to maximize $\gamma_{e2e}^{\mathrm{SC}}$ and/or $\gamma_{e2e}^{\mathrm{MRC}}$. Consequently, the optimal $\alpha^{⋆}$ is expressed as in following lemma.

**Lemma** **1.** *The closed-form expressions of optimal time-switching factor $\alpha^{⋆}$ corresponding to SC and MRC are given as:*

(27) $$\begin{array}{c} \alpha^{⋆}=\left\{\begin{array}{c} \alpha_{\mathrm{SC}}^{⋆}=\frac{1}{1+\eta \sqrt{\Phi XZT}},\\ \alpha_{\mathrm{MRC}}^{⋆}=\frac{1}{1+\eta \sqrt{\Phi YZT}}, \end{array}\right. \end{array}$$

**Proof.**  Since we consider DF protocol in the system model; thus, $\alpha^{⋆}$ can be obtained by solving following equation [42]

(28) $$\begin{array}{c} \gamma_{R}=\left\{\begin{array}{c} \gamma_{D_{m}}^{\mathrm{SC}},\;\mathrm{with}\;\mathrm{SC},\;\\ \gamma_{D}^{\mathrm{MRC}},\;\mathrm{with}\;\mathrm{MRC}, \end{array}\right. \end{array}$$

From (5), (7), and (12), the optimal $\alpha^{⋆}$ can be obtained as in Lemma 1. □

#### 3.2.1. With SC

By substituting $\alpha_{\mathrm{SC}}^{⋆}$ into (5) and using (14), ${\mathrm{OP}}_{\mathrm{SC}}^{⋆}$ can be expressed as

(29) $$\begin{array}{c} {\mathrm{OP}}_{\mathrm{SC}}^{⋆}=Pr\left(\sqrt{\frac{\Phi XT}{Z}}<\gamma_{\mathrm{th}}\right)=Pr\left(XT<\frac{\gamma_{\mathrm{th}}^{2}Z}{\Phi }\right)=\int_{0}^{\infty }F_{\varphi }\left(\frac{\gamma_{\mathrm{th}}^{2}z}{\Phi }\right)f_{Z}\left(z\right)dz, \end{array}$$

where φ≜XT.

**Lemma** **2.** *The CDF of φ can be expressed as*

(30) $$\begin{array}{c} F_{\varphi }\left(a\right)=1-2\sum_{m=0}^{M-1}{\left(-1\right)}^{m}C_{M-1}^{m}M\sqrt{\frac{\lambda_{\mathrm{SR}}\lambda_{\mathrm{RD}}a}{\left(m+1\right)}}K_{1}\left(2\sqrt{\lambda_{\mathrm{SR}}\lambda_{\mathrm{RD}}a\left(m+1\right)}\right). \end{array}$$

**Proof.**  From (29), CDF of φ is computed by

(31) $$\begin{array}{c} F_{\varphi }\left(a\right)=Pr\left(\varphi <a\right)=Pr\left(XT<a\right)=\int_{0}^{+\infty }F_{T}\left(\frac{a}{x}\right)f_{X}\left(x\right)dx, \end{array}$$

Then, using $f_{X}\left(x\right)$ expression from (10), $F_{\varphi }\left(a\right)$ is rewritten as

(32) $$\begin{array}{c} F_{\varphi }\left(a\right)=1-\sum_{m=0}^{M-1}{\left(-1\right)}^{m}C_{M-1}^{m}M\int_{0}^{+\infty }\lambda_{\mathrm{RD}}e^{-\lambda_{\mathrm{RD}}\left(m+1\right)x-\frac{\lambda_{\mathrm{SR}}a}{x}}dx, \end{array}$$

By adopting [41], $F_{\varphi }\left(a\right)$ can be obtained as in (30), which finishes the proof. □

**Theorem** **3.** *In dynamic time-switching factor scheme with SC, the closed-form expression of OP at the destination D can be expressed as*

(33) $$\begin{array}{c} {\mathrm{OP}}_{\mathrm{SC}}^{*}=1-\sum_{m=0}^{M-1}\frac{{\left(-1\right)}^{m}C_{M-1}^{m}M}{\left(m+1\right)}\exp\left(\frac{\gamma_{\mathrm{th}}^{2}\chi_{1}}{2\lambda_{\mathrm{SR}}}\right)W_{-1,\frac{1}{2}}\left(\frac{\gamma_{\mathrm{th}}^{2}\chi_{1}}{\lambda_{\mathrm{RR}}}\right). \end{array}$$

*where W{•} is the Whittaker function.*

**Proof.**  By applying Lemma 2, ${\mathrm{OP}}_{\mathrm{SC}}^{⋆}$ can be calculated as

(34) $$\begin{array}{cc} {\mathrm{OP}}_{\mathrm{SC}}^{*} & =1-2\sum_{m=0}^{M-1}{\left(-1\right)}^{m}C_{M-1}^{m}M\lambda_{\mathrm{RR}}\gamma_{\mathrm{th}}\int_{0}^{\infty }\exp\left(-\lambda_{\mathrm{RR}}z\right)\sqrt{z\chi_{2}}K_{1}\left(2\gamma_{\mathrm{th}}\sqrt{z\chi_{1}}\right)dz. \end{array}$$

By adopting [41], ${\mathrm{OP}}_{\mathrm{SC}}^{⋆}$ can be obtained as in (33), which finishes the proof. □

#### 3.2.2. With MRC

Similar to the above discussions, ${\mathrm{OP}}_{\mathrm{MRC}}^{⋆}$ is represented as

(35) $$\begin{array}{c} {\mathrm{OP}}_{\mathrm{MRC}}^{⋆}=Pr\left(\sqrt{\frac{\Phi YT}{Z}}<\gamma_{\mathrm{th}}\right)=\int_{0}^{\infty }F_{\tilde{\varphi }}\left(\frac{\gamma_{\mathrm{th}}^{2}z}{\Phi }\right)f_{Z}\left(z\right)dz, \end{array}$$

where $\tilde{\varphi }=YT$.

**Lemma** **3.** *The CDF of $\tilde{\varphi }$ can be expressed as*

(36) $$\begin{array}{c} F_{\tilde{\varphi }}\left(a\right)=1-\frac{2}{(M-1)!}{\left(\lambda_{\mathrm{SR}}\lambda_{\mathrm{RD}}a\right)}^{M/2}K_{M}\left(2\sqrt{\lambda_{\mathrm{SR}}\lambda_{\mathrm{RD}}a}\right). \end{array}$$

**Proof.**  First, the CDF of $\tilde{\varphi }$ can be calculated by

(37) $$\begin{array}{c} F_{\tilde{\varphi }}\left(a\right)=Pr\left(\tilde{\varphi }<a\right)\;=\;Pr\left(YT<a\right)\;=\;\int_{0}^{\infty }F_{T}\left(\frac{a}{y}\right)f_{Y}\left(y\right)dy, \end{array}$$

Then, by substituting (13) into (37), we have

(38) $$\begin{array}{c} F_{\tilde{\varphi }}\left(a\right)=1-\int_{0}^{\infty }\frac{{\left(\lambda_{\mathrm{RD}}\right)}^{M}}{(M-1)!}y^{M-1}\exp\left(-\lambda_{\mathrm{RD}}y-\frac{\lambda_{\mathrm{SR}}a}{y}\right)dy, \end{array}$$

By adopting [41], $F_{\tilde{\varphi }}$ can be obtained as in Lemma 3. □

**Theorem** **4.** *In dynamic time-switching factor scheme with MRC, the closed-form expression of OP at the destination D can be expressed as*

(39) $$\begin{array}{cc} {\mathrm{OP}}_{\mathrm{MRC}}^{⋆}\; & =1-\frac{{\left(\gamma_{\mathrm{th}}\right)}^{M-1}}{(M-1)!}\times {\left(\frac{\chi_{3}}{\lambda_{\mathrm{RR}}}\right)}^{\frac{M}{2}-\frac{1}{2}}\Gamma \left(M+1\right)\\ \; & \times \exp\left(\frac{\gamma_{\mathrm{th}}^{2}\chi_{3}}{2\lambda_{\mathrm{RR}}}\right)\times W_{-\frac{M}{2}-\frac{1}{2},\frac{M}{2}}\left(\frac{\gamma_{\mathrm{th}}^{2}\chi_{3}}{\lambda_{\mathrm{RR}}}\right), \end{array}$$

*where Γ{•} is the Gamma function.*

**Proof.**  Using $F_{\tilde{\varphi }}\left(a\right)$ obtained in (36), ${\mathrm{OP}}_{\mathrm{MRC}}^{⋆}$ can be computed as

(40) $$\begin{array}{c} {\mathrm{OP}}_{\mathrm{MRC}}^{⋆}=1-\frac{2{\left(\gamma_{\mathrm{th}}\right)}^{M}}{(M-1)!}\int_{0}^{\infty }\lambda_{\mathrm{RR}}{\left(\frac{z\chi_{3}}{\Phi }\right)}^{M/2}\exp\left(-\lambda_{\mathrm{RR}}z\right)K_{M}\left(2\gamma_{\mathrm{th}}\sqrt{z\chi_{3}}\right)dz, \end{array}$$

Finally, By adopting [41], ${\mathrm{OP}}_{\mathrm{MRC}}^{⋆}$ can be obtained as in (39), which finishes the proof of Theorem 4. □

## 4. Simulation Results

In this section, the analytical model developed in Section 3 is implemented in Matlab to evaluate two proposed EH DF relaying systems’ performance. Specifically, Monte Carlo simulations are used to verify the correctness of theoretical derivations. To obtain the outage probability for proposed schemes, we perform $10^{6}$ independent trials, and in each trial, we create Rayleigh fading channels for all the links [43,44]. The simulated parameters are listed in Table 1. Unless otherwise stated, we assume that the SNR threshold is set as $\gamma_{\mathrm{th}}=1$, energy harvesting efficiency η=0.8, the number of antennas at the destination M=2 or 3, the transmit-power-to-noise-ratio Ψ= 5 dB, and the static time-switching factor α is set to 0.25, 0.5, or 0.75. In the simulation environment, we consider a Cartesian coordinate system, in which the distances between S→R and R→D are respectively set as $d_{\mathrm{SR}}$ = 1, $d_{\mathrm{RD}}$ = 0.5. Furthermore, we consider a simplified path-loss model, i.e., $\lambda_{I}=d_{I}^{\nu }$, where I∈{SR,RD} and ν is the path-loss exponent. Without loss of generality, we present all simulation results and exactly theoretical results by markers and solid lines, respectively.

**Figure 3:**
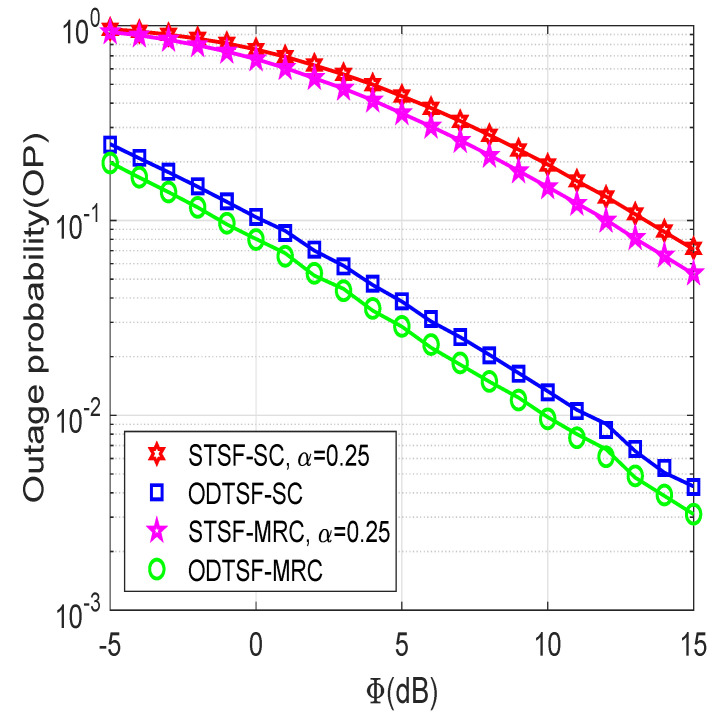
Outage probability versus Φ (in dB) with $\gamma_{\mathrm{th}}=1$ bps/Hz, η=0.8, *M* = 2.

Figure 3 presents OP as a function of Φ (dB), with η=0.8, $\gamma_{\mathrm{th}}=1$, and M=2. In Figure 3, we compare ODTSF with STSF, whereas each scheme is considered in both SC and MRC. It can be observed that the ODTSF obtains better outage performance than STSF with α=0.25. It is expected since the proposed ODTSF can acquire the optimal value of $\alpha^{⋆}$ to maximize the end-to-end SNR at the destination, which is mentioned in Lemma 1. Thus, the achievable data rate at the destination of ODTSF is better than that of STSF. Consequently, it has more chance to decode the received signal successfully and has a better outage result. It is also observed that the increasing Φ value, i.e., Φ from −5 to 15 dB, leads to the improvement of OP. This is plausible because the higher the Φ is, the better the SNR can obtain mentioned in (7) and (12). Moreover, the performance of MRC-based methods is better than that compared to SC-based methods. This is because the destination D can incorporate received signals from all antennas in MRC, which improves the total throughput. D only selects the best channel when it applies SC. From the above discussions, it is easy to see that the ODTSF-MRC achieves the best results as compared with others.

**Figure 4:**
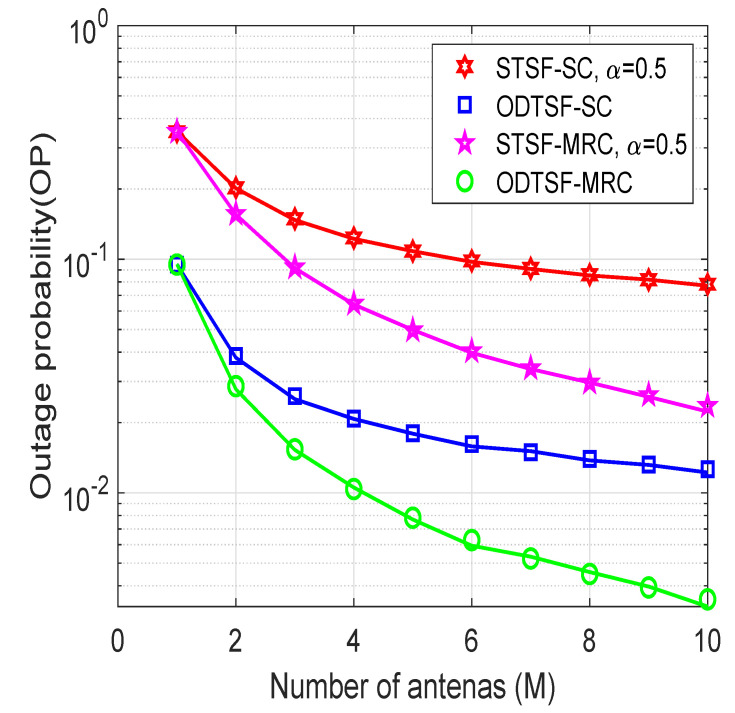
Outage probability versus number of antennas at the destination with $\gamma_{\mathrm{th}}=1$ bps/Hz, η=0.8, Φ=5 dB.

In Figure 4, the outage probability is plotted as a function of the number of antennas at the destination, where η=0.8, $\gamma_{\mathrm{th}}=1$, and Φ=5 dB. It can be seen that the outage performance is enhanced with a higher number of antennas at the destination, i.e., *M* varies from 1 to 10. This can be explained that by increasing the number of antennas, we have more choices to choose the best antenna as in SC, which enhances the destination’s rate. Furthermore, in MRC, the higher the *M* value is, the more the throughput can be obtained. This is because the destination can combine signals from all antennas in MRC. One more notable point in Figure 4 is that the gap between MRC-based and SC-based methods is larger with a higher number of antennas *M*. This means that *M* value has more impact on MRC-based methods compared to SC-based ones.

**Figure 5:**
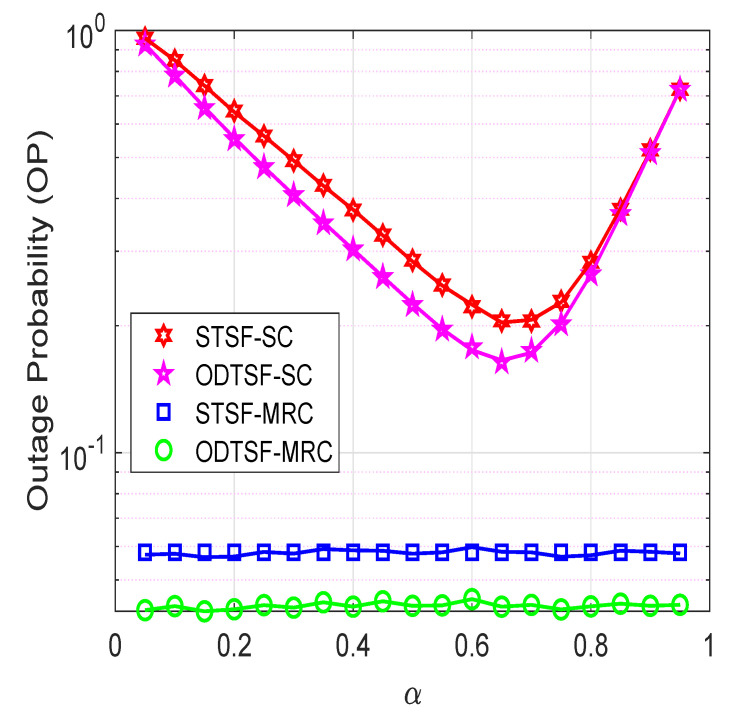
Outage probability versus time-switching factor α with $\gamma_{\mathrm{th}}=1$ bps/Hz, η=0.8, Φ=3 dB, *M* = 2.

In Figure 5, we investigated influences of the time-switching factor α on the outage probability, where η=0.8, $\gamma_{\mathrm{th}}=1$, M=2, and Φ= 3 dB. The α value plays a crucial role in relaying systems because it not only affects the harvested energy at the relay R but also the data rate at the destination. It can be illustrated from Figure 2 that the relay R can harvest more energy with a higher value of α, but less time is allocated to transmit data R→D. This explains the fact that the STSF-SC and STSF-MRC can obtain the best value at optimal α, then its performance decreases. Notably, the ODTSF always has better outage performance than STSF because this scheme uses the optimal α value while designing the system. This also explains the fact that the ODTSF values do not change when we vary α values from 0.05 to 0.95. Similar to Figure 3 and Figure 4, the ODTSF-MRC obtains the best performance as compared to others.

**Figure 6:**
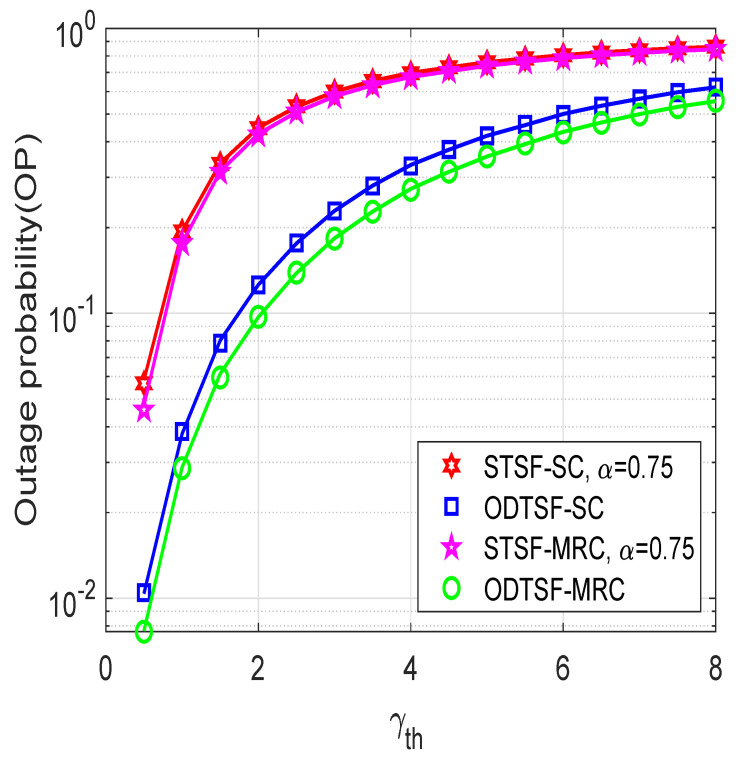
Outage probability versus $\gamma_{\mathrm{th}}$ with η=0.8, *M* = 2, and Φ=5 dB.

In Figure 6, we illustrate the outage probability as a function of the SNR threshold $\gamma_{\mathrm{th}}$, where η=0.8, M=2, and Φ=5 dB. First, we observed that all schemes have worse performance as $\gamma_{\mathrm{th}}$ value increases. This is because the higher the SNR threshold is, the less chance the destination can successfully decode the signal, which is shown in (14). Moreover, when $\gamma_{\mathrm{th}}$ value is large enough, the outage probability of all schemes converges to a saturation value, i.e., OP equals 1. In Figure 3, Figure 4, Figure 5 and Figure 6, the simulation agrees with the theoretical analysis results, which confirms our mathematical derivations. From the simulations, we conclude that the ODTSF-MRC is the best option to design the system. In the case that our system prefers to use SC at the destination, the OTDSF-SC becomes a right choice.

## 5. Conclusions

In this paper, we have investigated the outage performance for a FD- and DF-based SWIPT relaying system consisting of a single-antenna source, a FD- and EH-enabled relay, and a multi-antenna destination. We proposed four novel relaying methods, namely STSF-SC, STSF-MRC, ODTSF-SC, and ODTSF-MRC to investigate the impact of FD and EH on the outage performance of a dual-hop cooperative relaying system. Moreover, we derive the exact closed-formed expression in terms of outage probability of four proposed protocols over i.i.d. Rayleigh block fading, where the relay can harvest energy from the source’s RF signals. The numerical results verified that the proposed ODTSF-MRC always obtains the best performance compared to other schemes. For future work, it is interesting to extend this work by considering non-orthogonal multiple access (NOMA), multiple sources/relays/destinations, or uses a UAV-assisted relay.

## Figures and Tables

**Table 1:** Simulation parameters.

Symbol	Parameter Name	Fixed Value	Varying Range
$\gamma_{\mathrm{th}}$	SNR threshold of the system	1	0.5 to 8
η	Energy harvesting efficiency	0.8	none
α	Time-switching factor	0.05–0.95	0.05 to 0.95
$d_{\mathrm{SR}}$	Distance between source S and relay R	1	none
$d_{\mathrm{RD}}$	Distance between relay R and destination D	0.5	none
$\lambda_{\mathrm{SR}}$	Rate parameter of $|h_{\mathrm{SR}}{|}^{2}$	1	none
$\lambda_{\mathrm{RD}}$	Rate parameter of $|h_{R}{|}^{2}$	0.5	none
$\lambda_{\mathrm{RR}}$	Rate parameter of $|h_{\mathrm{RR}}{|}^{2}$	5	none
Ψ	Transmit-power-to-noise-ratio	5 dB	−5 to 15 (dB)
*M*	No. of antennas at the destination	2; 3	1 to 10

## Data Availability

Not applicable.
